# Synchronous Gastric Signet-Ring Cell Adenocarcinoma and Mucosa-Associated Lymphoid Tissue (MALT) Lymphoma Treated With Robotic Total Gastrectomy: A Case Report and Literature Review

**DOI:** 10.7759/cureus.115251

**Published:** 2026-08-26

**Authors:** Alexandre Gomes, Alexandre Ferreira Duarte, Victor A Duarte

**Affiliations:** 1 Department of Surgery, Faculdade de Ciências Médicas e da Saúde, Pontifícia Universidade Católica de São Paulo (PUCSP), São Paulo, BRA; 2 Department of Colorectal Surgery, Unimed Medical Institute, São Paulo, BRA; 3 Department of Gastrointestinal Surgery, Universidade Federal de São Paulo, Sorocaba, BRA

**Keywords:** gastric adenocarcinoma, gastric malt lymphoma, helicobacter pylori, robotic gastrectomy, signet-ring cell carcinoma, synchronous neoplasms

## Abstract

The synchronous occurrence of gastric adenocarcinoma and mucosa-associated lymphoid tissue (MALT) lymphoma is rare, and coexistence with signet-ring cell adenocarcinoma is particularly uncommon. We report a 43-year-old White Brazilian man with a history of gastroesophageal reflux disease, nondysplastic Barrett’s esophagus, and previous laparoscopic Nissen fundoplication. During endoscopic follow-up, chronic gastritis, persistent *Helicobacter pylori* (*H. pylori*) infection, and gastric MALT lymphoma were identified. Despite eradication therapy, *H. pylori* and the lymphomatous infiltrate persisted, and a 15-mm depressed antral lesion became evident. Serial biopsies and immunohistochemistry demonstrated synchronous signet-ring cell adenocarcinoma and MALT lymphoma. Endoscopic ultrasonography suggested focal superficial submucosal involvement, whereas computed tomography and positron emission tomography-computed tomography showed no metastatic disease. Following multidisciplinary review, the patient underwent robotic total gastrectomy with D2 lymphadenectomy and Roux-en-Y reconstruction using the da Vinci X platform. The postoperative course was uneventful, and discharge occurred on postoperative day 7. Surgical pathology revealed a 1.2-cm poorly differentiated signet-ring cell adenocarcinoma confined to the muscularis mucosae, residual MALT lymphoma, persistent *H. pylori*, and no metastatic tumor in 19 lymph nodes (pT1aN0M0). This case emphasizes the need for systematic endoscopic mapping and comprehensive histopathological assessment in gastric MALT lymphoma. In a surgically altered upper abdomen with dense adhesions and potential compromise of remnant fundic perfusion, robotic total gastrectomy provided a feasible minimally invasive strategy for treating both neoplasms.

## Introduction

Synchronous primary malignancies are distinct neoplasms diagnosed simultaneously or within a short predefined interval, commonly within six months [1]. Multiple synchronous gastric neoplasms have been reported in 4.4-10.9% of patients with gastric cancer. However, the coexistence of gastric adenocarcinoma and mucosa-associated lymphoid tissue (MALT) lymphoma accounts for only a small fraction of these cases [2,3]. The published evidence consists primarily of case reports and small retrospective series, and the true incidence is probably underestimated because one component may be detected only after extensive examination of a resection specimen [1,4].

Chronic *Helicobacter pylori* (*H. pylori*) infection provides a plausible shared biological substrate. Antigen-driven B-cell proliferation contributes to gastric MALT lymphomagenesis, whereas chronic epithelial injury promotes gastric carcinogenesis. Nevertheless, diffuse-type and signet-ring cell cancers may follow molecular pathways that differ from the conventional atrophy-metaplasia-dysplasia sequence [5,6,7]. Collision or composite tumors containing both MALT lymphoma and signet-ring cell carcinoma have been described only rarely [8-10].

We present a patient in whom both neoplasms were diagnosed preoperatively after serial endoscopic examinations and biopsies. The case also illustrates the limitations of endoscopic ultrasonography (EUS) for staging an ulcerated early gastric lesion and the technical rationale for robotic total gastrectomy after previous fundoplication with hiatal mesh reinforcement.

At the most recent follow-up, approximately five months after surgery, the patient remained clinically well, without evidence of recurrent adenocarcinoma or MALT lymphoma.

## Case presentation

A 43-year-old White Brazilian man with long-standing typical gastroesophageal reflux symptoms was evaluated and treated at Hospital Unimed de Sorocaba in Sorocaba, São Paulo, Brazil. He underwent upper gastrointestinal endoscopy approximately three years before the most recent follow-up. The examination showed a 2-cm hiatal hernia and nondysplastic Barrett’s esophagus; testing for *H. pylori* was negative. He reported no relevant comorbidities or family history of malignancy and denied smoking and alcohol use.

Approximately two years and two months before the most recent follow-up, he underwent laparoscopic Nissen fundoplication with hiatal reinforcement using a lightweight, large-pore mesh (Ultrapro®, Ethicon, Inc., Johnson & Johnson, Raritan, NJ, USA). His reflux symptoms resolved. Surveillance endoscopy approximately two years before the most recent follow-up showed an intact fundoplication with a competent Hill grade I flap valve and no relevant gastric abnormality.

**Figure 1:**
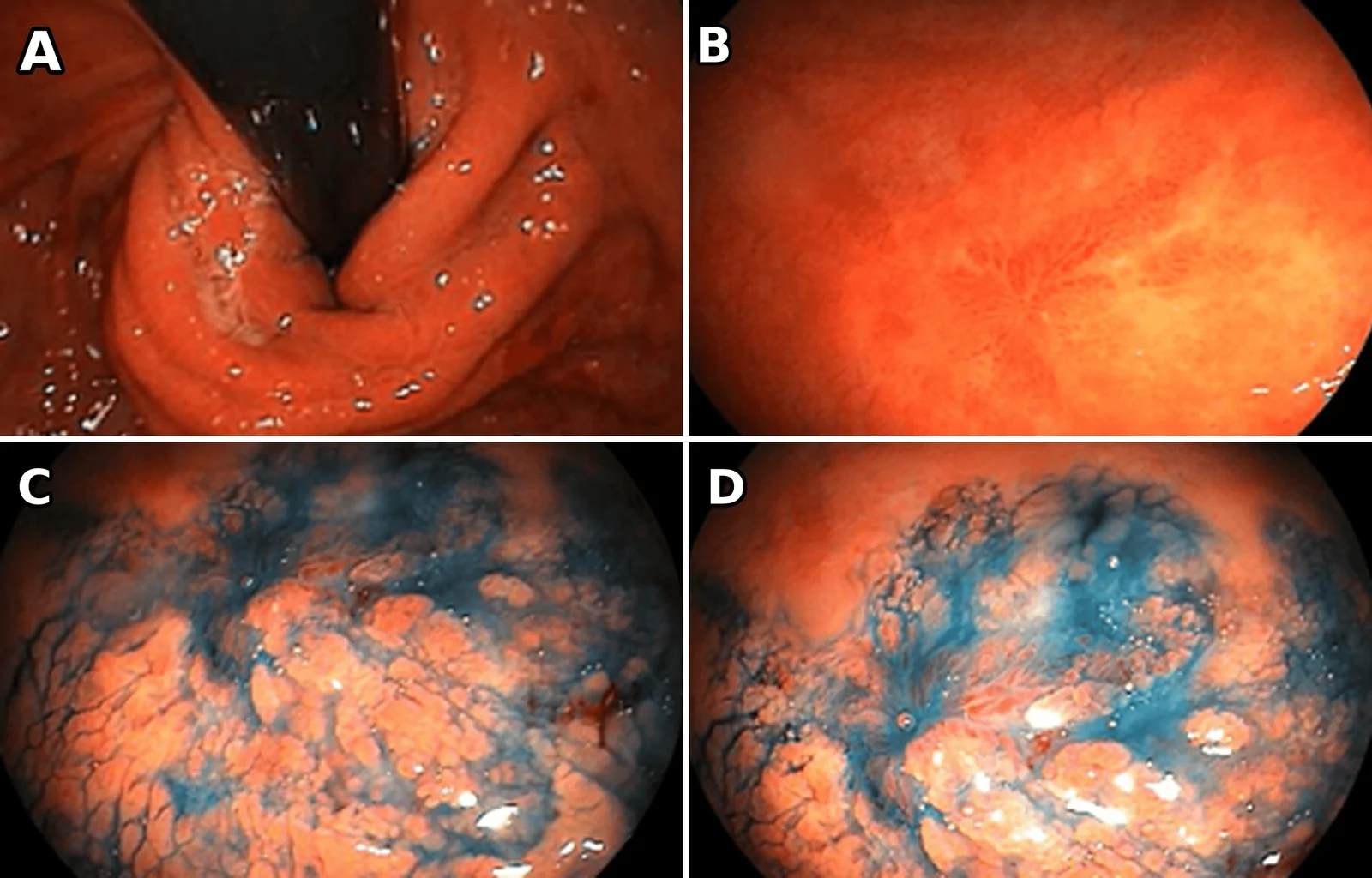
Upper gastrointestinal endoscopy showing (A) previous fundoplication, (B) irregular antral mucosa at the biopsy site, and (C-D) indigo carmine chromoendoscopy highlighting the antral mucosal irregularity

Approximately one year before the most recent follow-up, endoscopy for dyspeptic symptoms revealed an intact fundoplication, moderate erythematous and friable pangastritis, and irregular, elevated areas with a small antral erosion (Figure 1).

**Figure 2:**
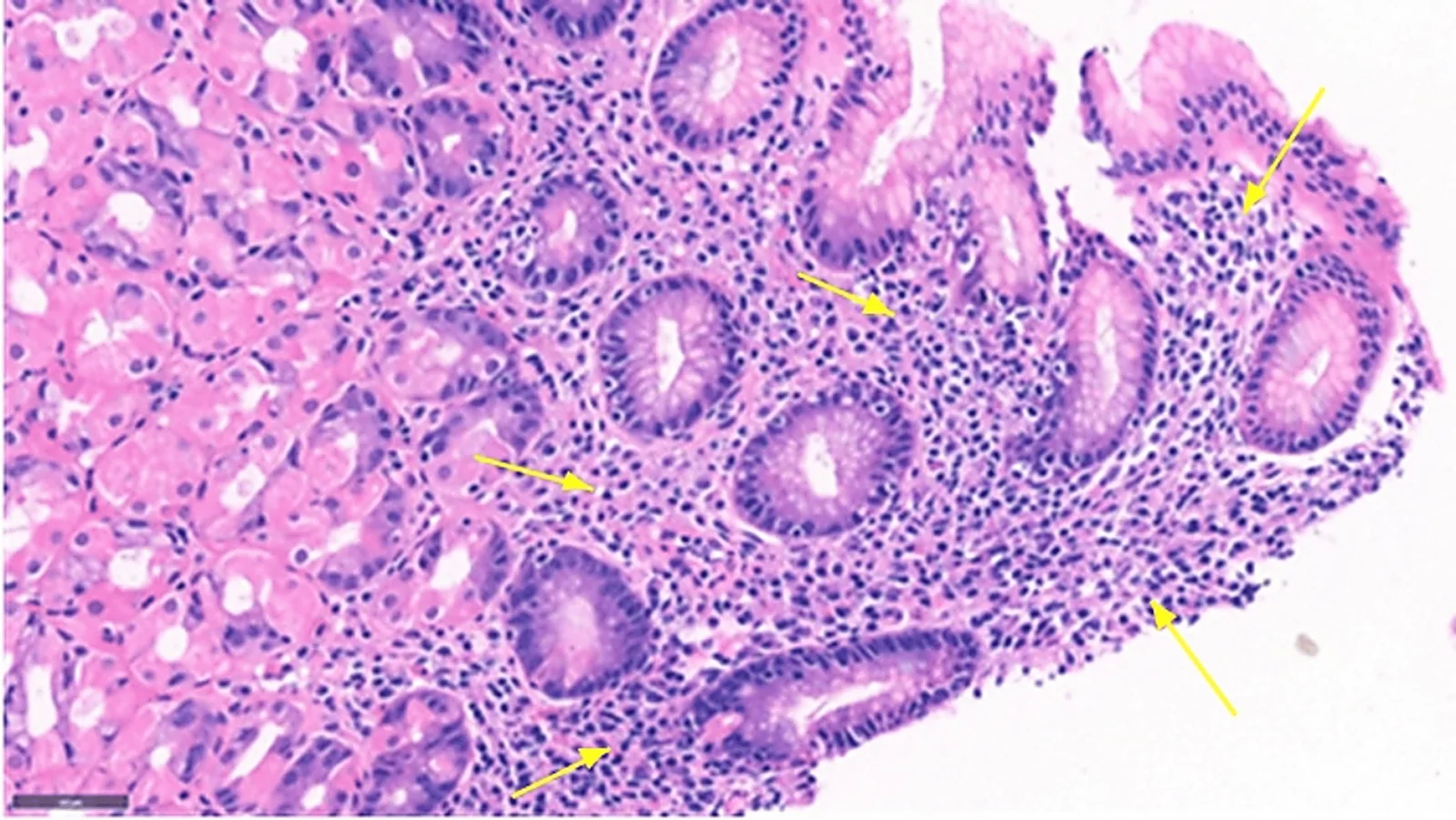
Gastric biopsy (hematoxylin and eosin, original magnification ×200) showing dense lymphoplasmacytic infiltrate with lymphoepithelial lesions (yellow arrows) consistent with MALT lymphoma MALT: mucosa-associated lymphoid tissue

**Figure 3:**
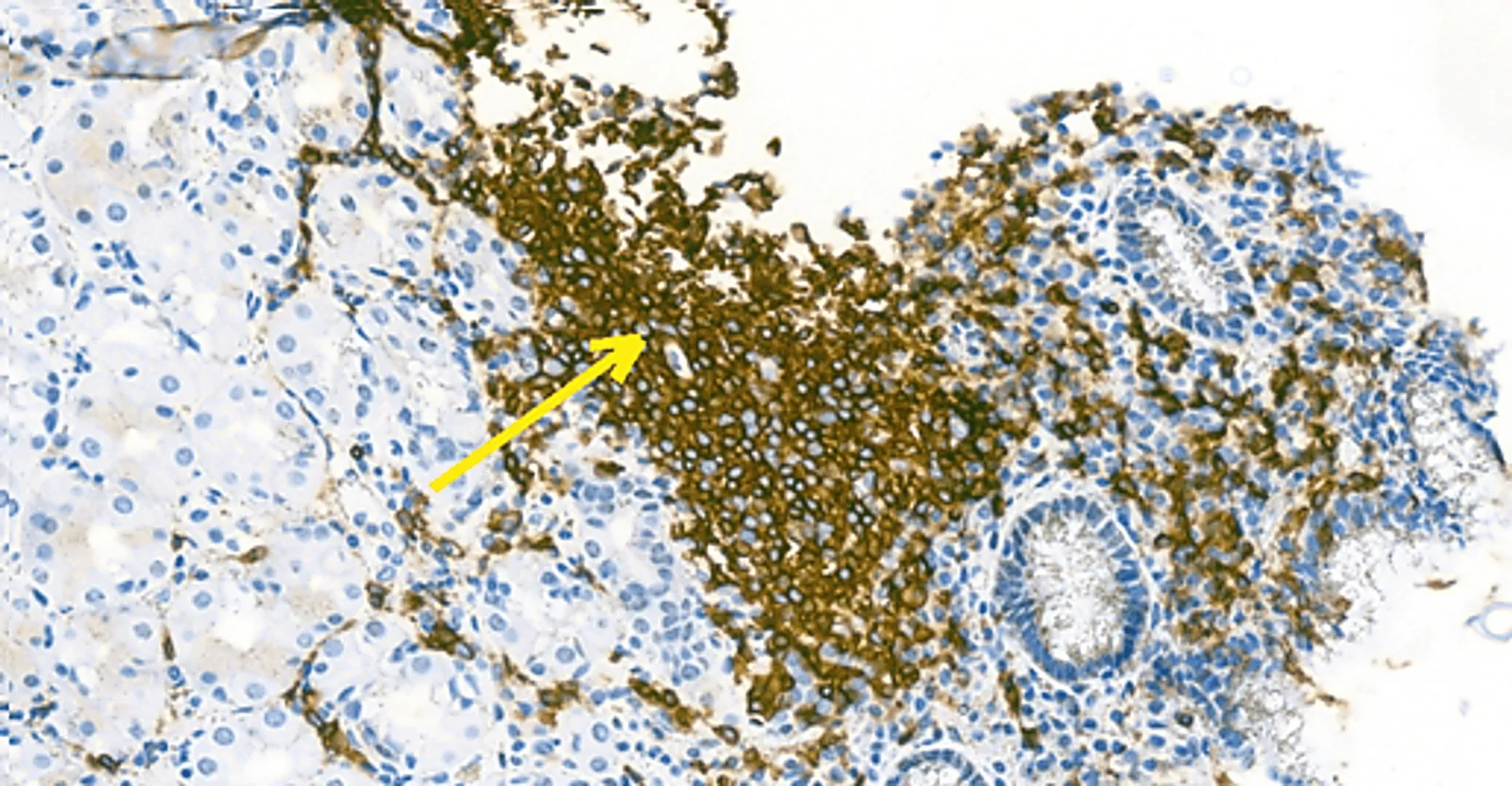
Immunohistochemistry (anti-CD20 antibody, original magnification ×200) showing diffuse B-cell lineage marker expression within the lymphoid infiltrate

Histology demonstrated chronic follicular gastritis with atypical lymphoid cells and *H. pylori*. Histology and immunohistochemistry supported a diagnosis of gastric MALT lymphoma (Figures 2-3, Table 1).

**Table 1:** Immunohistochemical panel from the initial gastric biopsy showing the combined findings supporting a predominantly B-cell lymphoid proliferation in gastric mucosa Interpretation was integrated with the histomorphology.

Antigen	Clone	Result
AE1/AE3	AE1/AE3	Positive in epithelium
BCL-2	124	Positive in numerous lymphocytes
CD10	SP67	Negative; internal control positive
CD138	B-A38	Positive in plasma cells
CD20	L26	Positive in numerous lymphocytes
CD23	CD23	Positive in rare follicular dendritic cells
CD3	2GV6	Positive in several lymphocytes
CD5	4C7	Positive in several lymphocytes
Cyclin D1	SP4-R	Negative; internal control positive
CDX2	DAK-CDX2	Negative
Kappa	Polyclonal	Positive in plasma cells
Ki-67	30-Sep	Positive in approximately 5% of lymphocytes
Lambda	Polyclonal	Positive in plasma cells
p53	DO-7	Negative

Staging computed tomography and positron emission tomography-computed tomography did not demonstrate distant or nodal disease.

**Figure 4:**
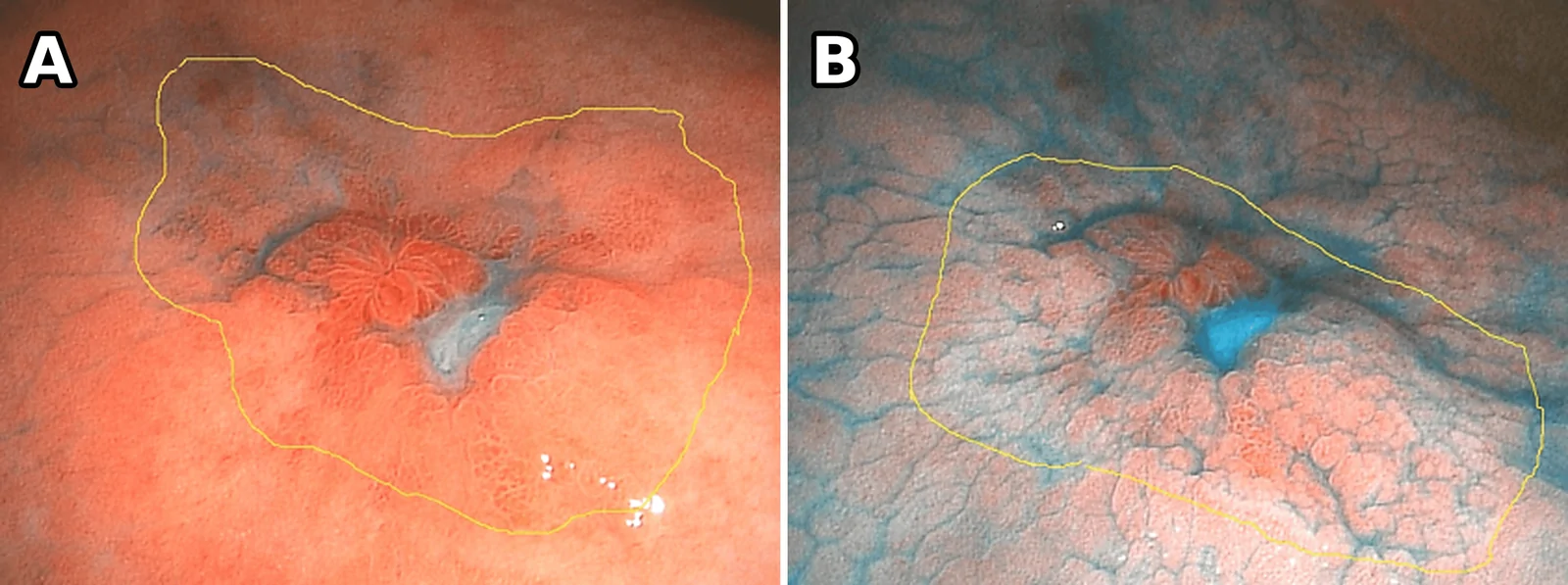
Superficial antral neoplasm (Paris 0-IIa+IIc), measuring approximately 15 mm, with a central depression and small fibrin-covered ulceration. (A, B) Chromoendoscopy enhances lesion demarcation and demonstrates surface architectural irregularity

*H. pylori* eradication was attempted with pantoprazole 40 mg, amoxicillin 1 g, and clarithromycin 500 mg, each administered twice daily for 14 days. Three months later, endoscopy showed persistent moderate pangastritis, persistent *H. pylori* infection, and a 15-mm slightly elevated lesion with a well-demarcated central depression and fibrin-covered ulceration in the antrum (Paris 0-IIa+IIc) (Figure 4).

Repeat biopsies disclosed poorly differentiated signet-ring cell adenocarcinoma together with MALT lymphoma. A second eradication regimen comprising a proton pump inhibitor, amoxicillin, and levofloxacin was prescribed before definitive surgery, but *H. pylori* remained detectable.

Endoscopic ultrasonography

**Figure 5:**
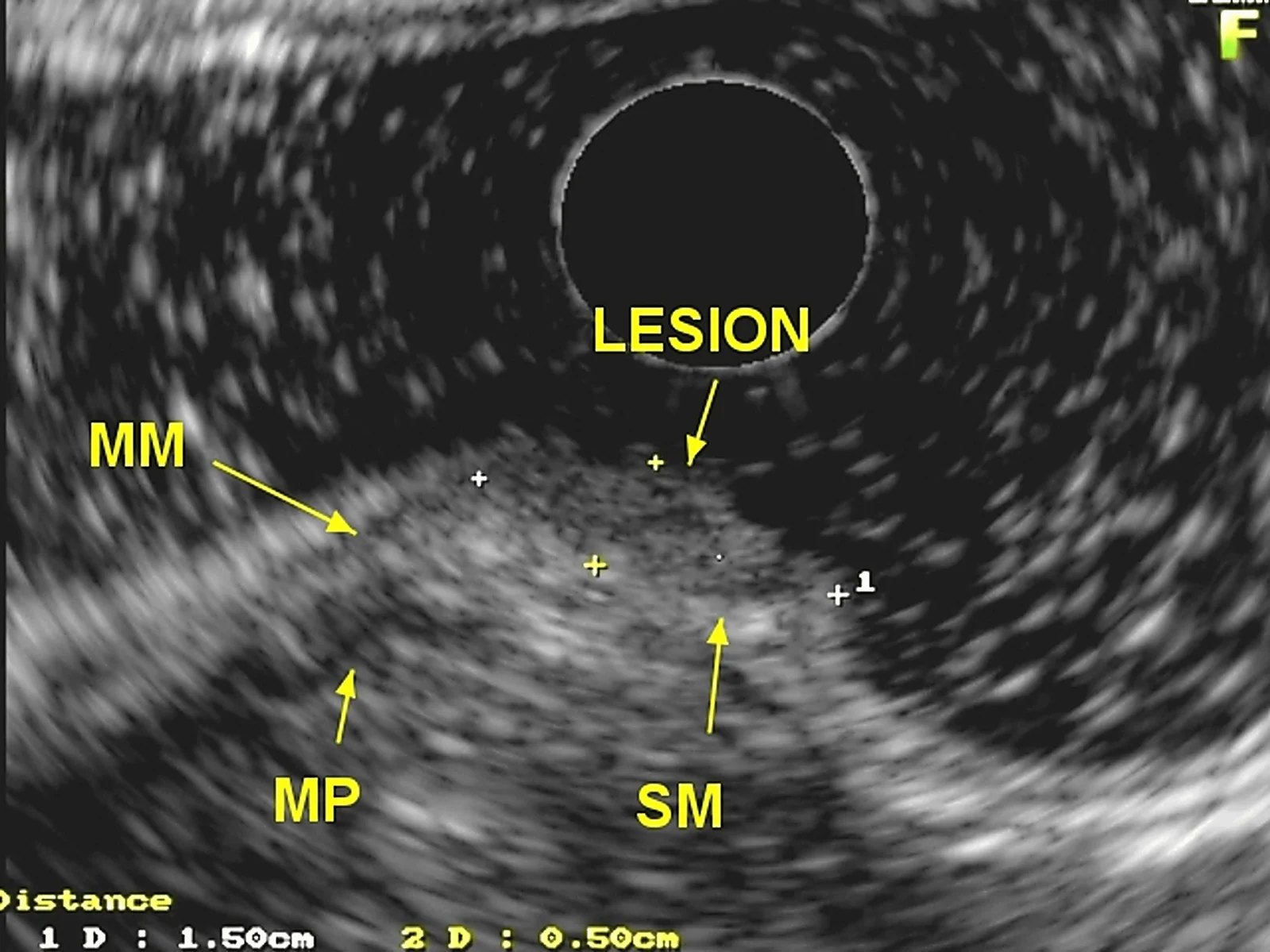
Radial EUS showing a hypoechoic gastric lesion involving the mucosa and MM, with focal contact with the SM; the MP is intact Electronic caliper measurements: 1.50 × 0.50 cm MM: muscularis mucosae, SM: superficial submucosa, MP: muscularis propria, EUS: endoscopic ultrasonography

Radial EUS (Fujinon EG-530UR2, FUJIFILM Corporation, Tokyo, Japan) demonstrated a heterogeneous hypoechoic lesion measuring approximately 15 × 5 mm. The lesion involved the mucosa and muscularis mucosae, with focal contact and minimal apparent extension into the superficial submucosa; the muscularis propria remained intact. The findings were interpreted as possible cT1b disease (Figure 5).

Endoscopic submucosal dissection was not selected because the lesion was an ulcerated, poorly differentiated signet-ring cell adenocarcinoma with suspected submucosal involvement and a synchronous lymphomatous component. After multidisciplinary discussion, total rather than subtotal gastrectomy was chosen. The previous fundoplication, hiatal mesh, and division of short gastric vessels were expected to produce dense proximal adhesions and potentially jeopardize fundic perfusion if a proximal remnant were retained.

**Figure 6:**
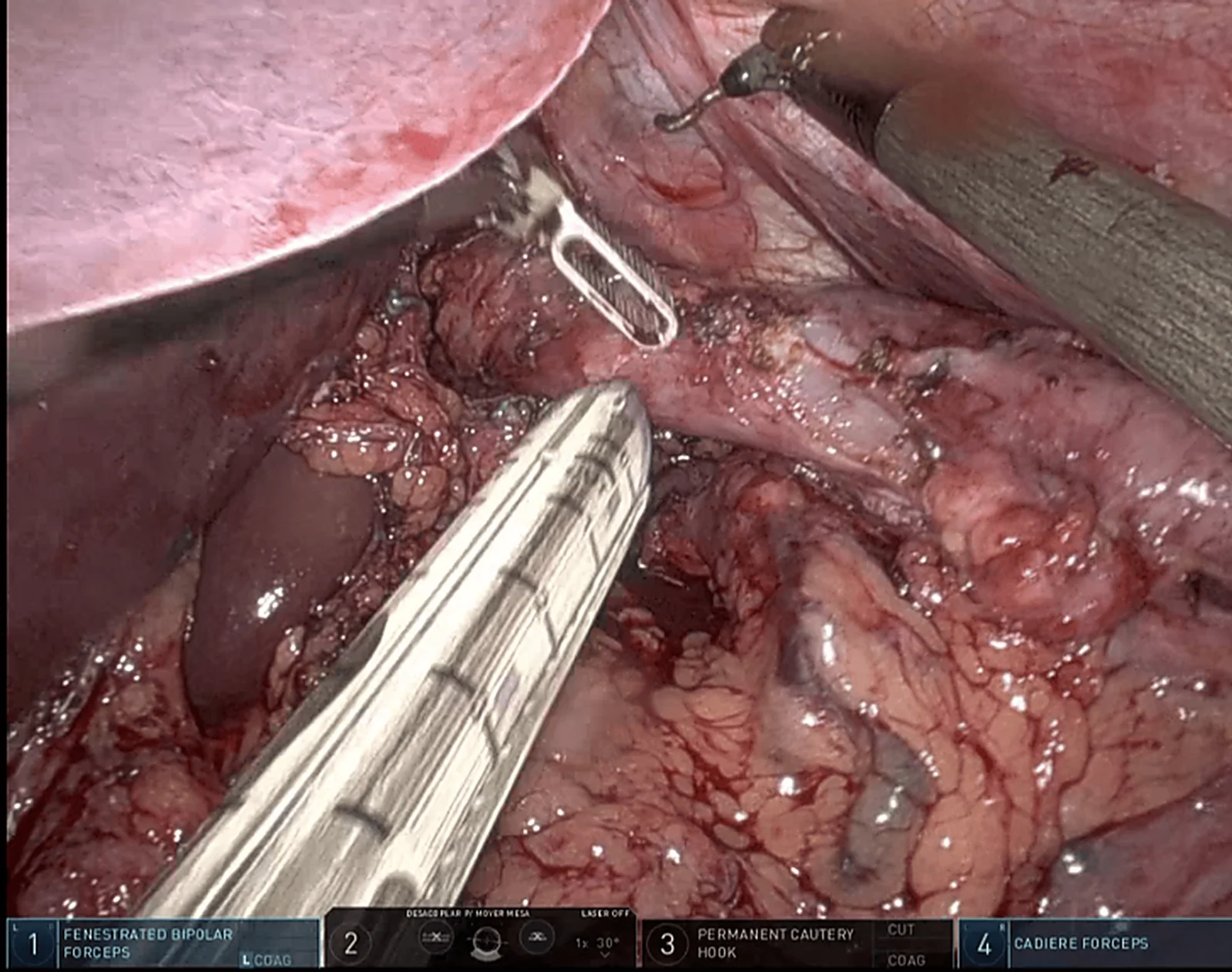
Robotic total gastrectomy showing an intraoperative view during esophageal transection at the esophagogastric junction

**Figure 7:**
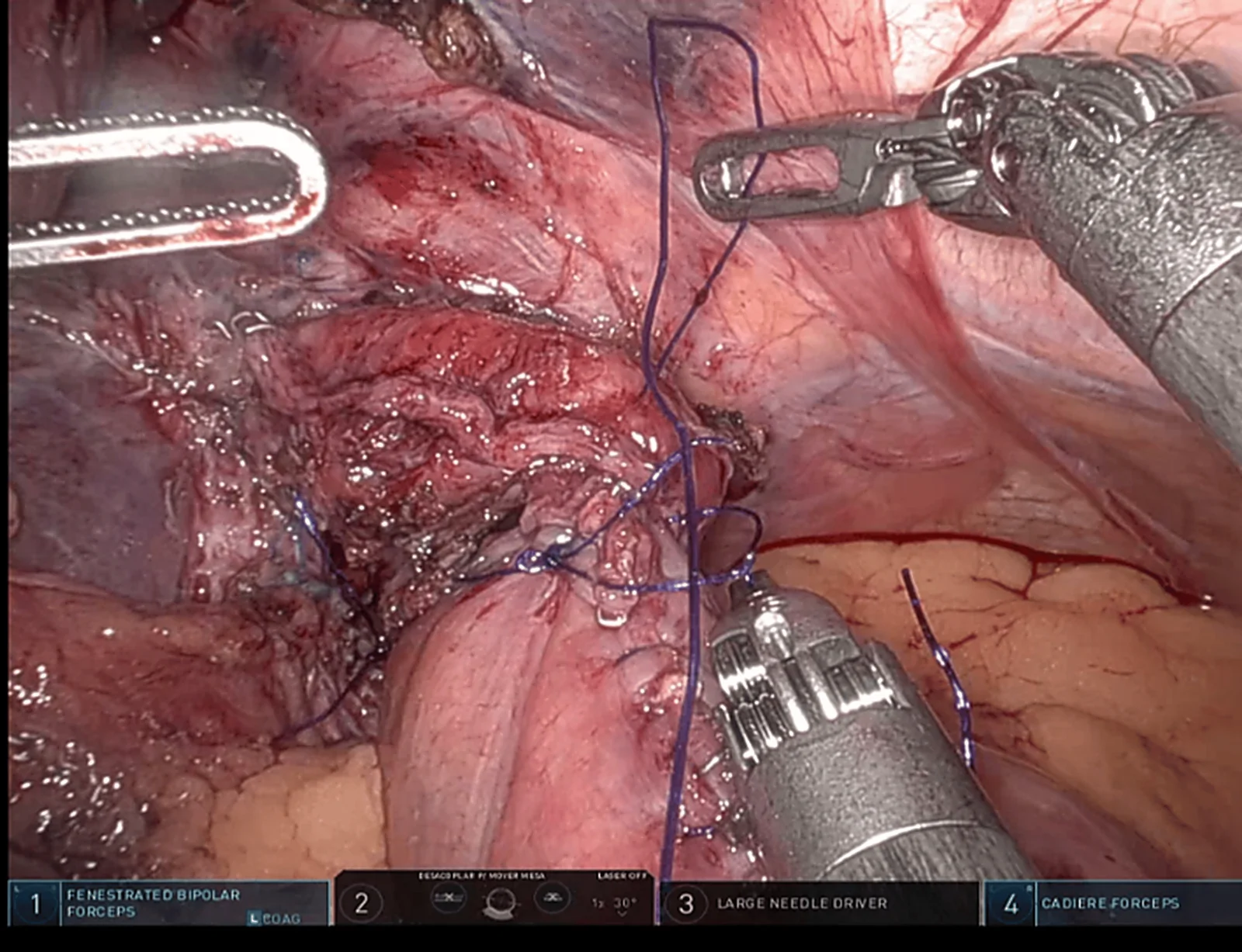
Robotic total gastrectomy showing an intraoperative view of the mechanically created esophagojejunal anastomosis before completion of the Roux-en-Y reconstruction

Approximately five months before the most recent follow-up, the patient underwent robotic total gastrectomy with D2 lymphadenectomy and Roux-en-Y reconstruction using the da Vinci X platform. The esophagojejunal anastomosis was created mechanically and reinforced with a continuous suture (Figures 6-7).

Console time was 275 minutes. The operation was completed without conversion to laparotomy. The postoperative course was uncomplicated, and the patient was discharged on postoperative day 7.

Surgical pathology

**Figure 8:**
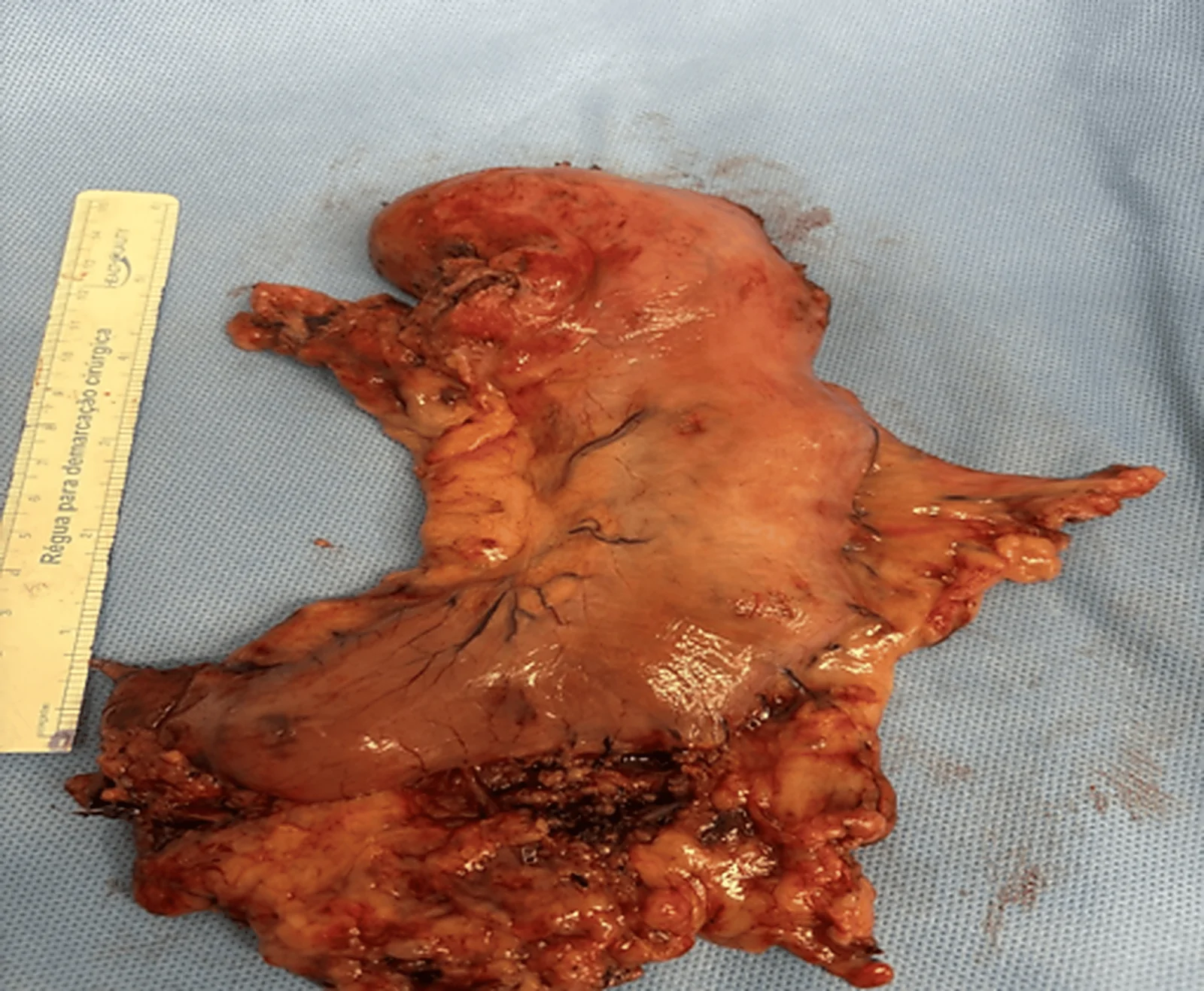
External (serosal) surface of the total gastrectomy specimen with a ruler for scale

**Figure 9:**
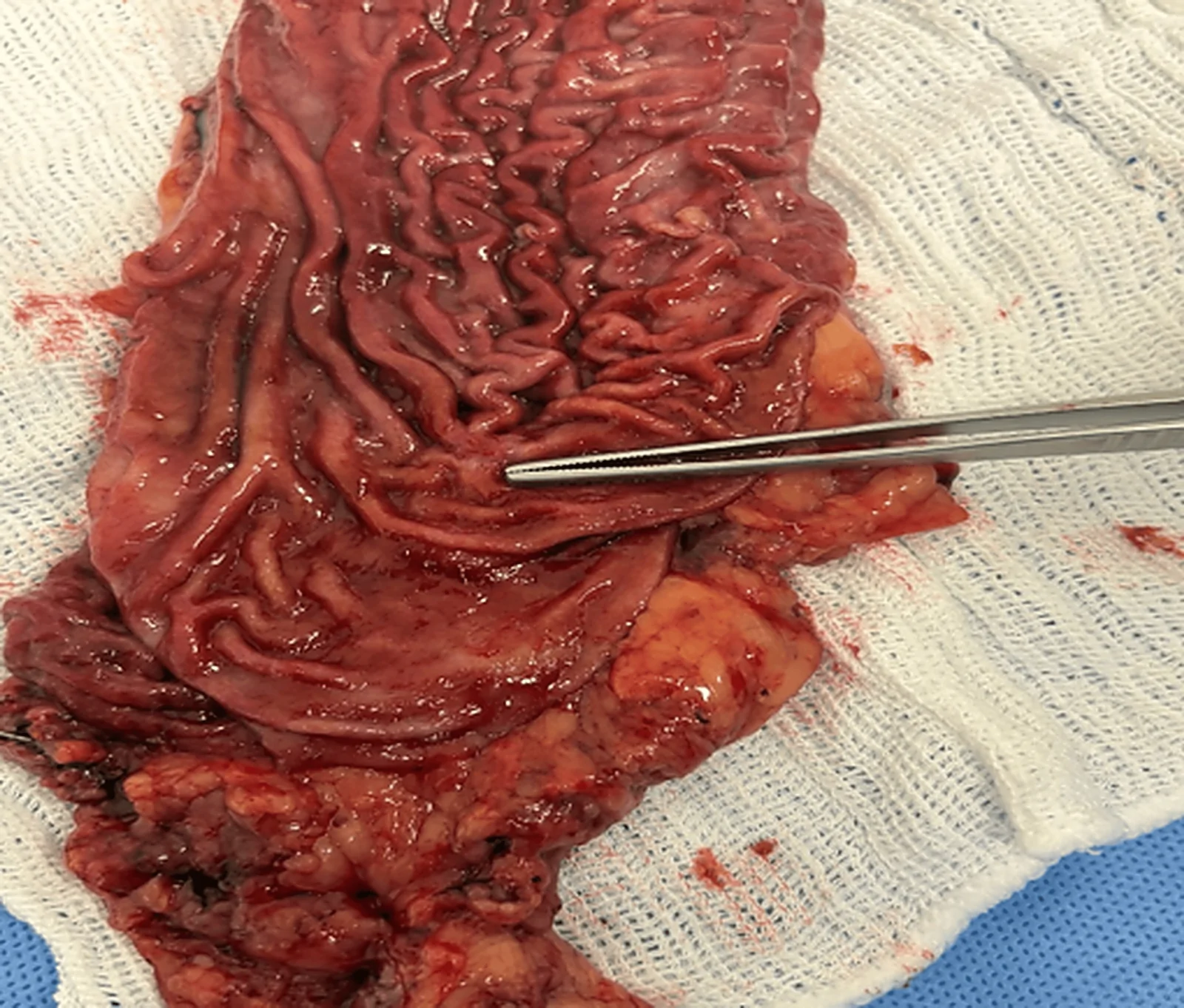
Open gastrectomy specimen showing the mucosal surface; the forceps indicate the tumor site

**Figure 10:**
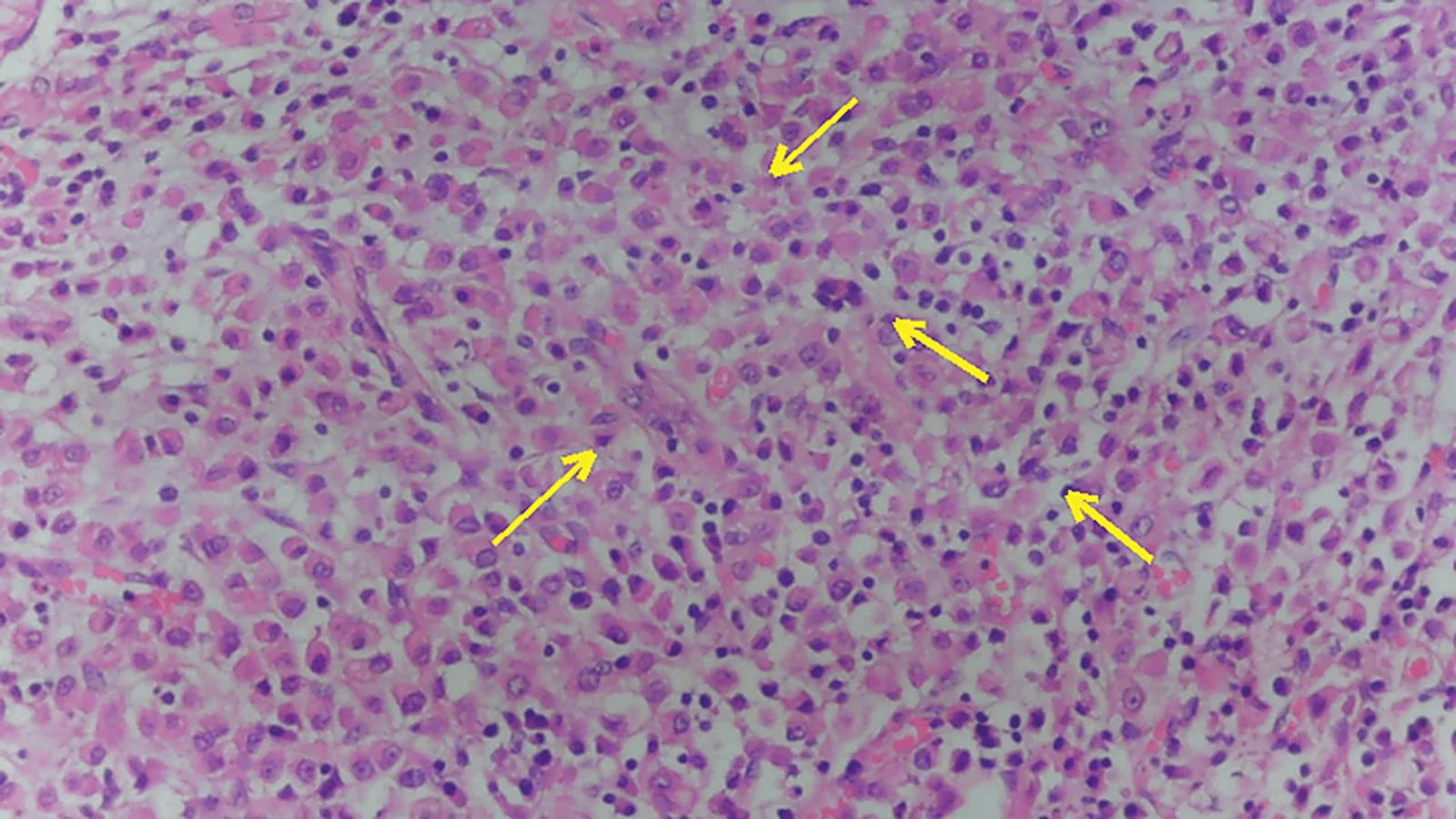
High-power hematoxylin and eosin image (original magnification ×400) showing poorly differentiated adenocarcinoma with signet-ring cells containing eccentric nuclei displaced by intracytoplasmic mucin

**Figure 11:**
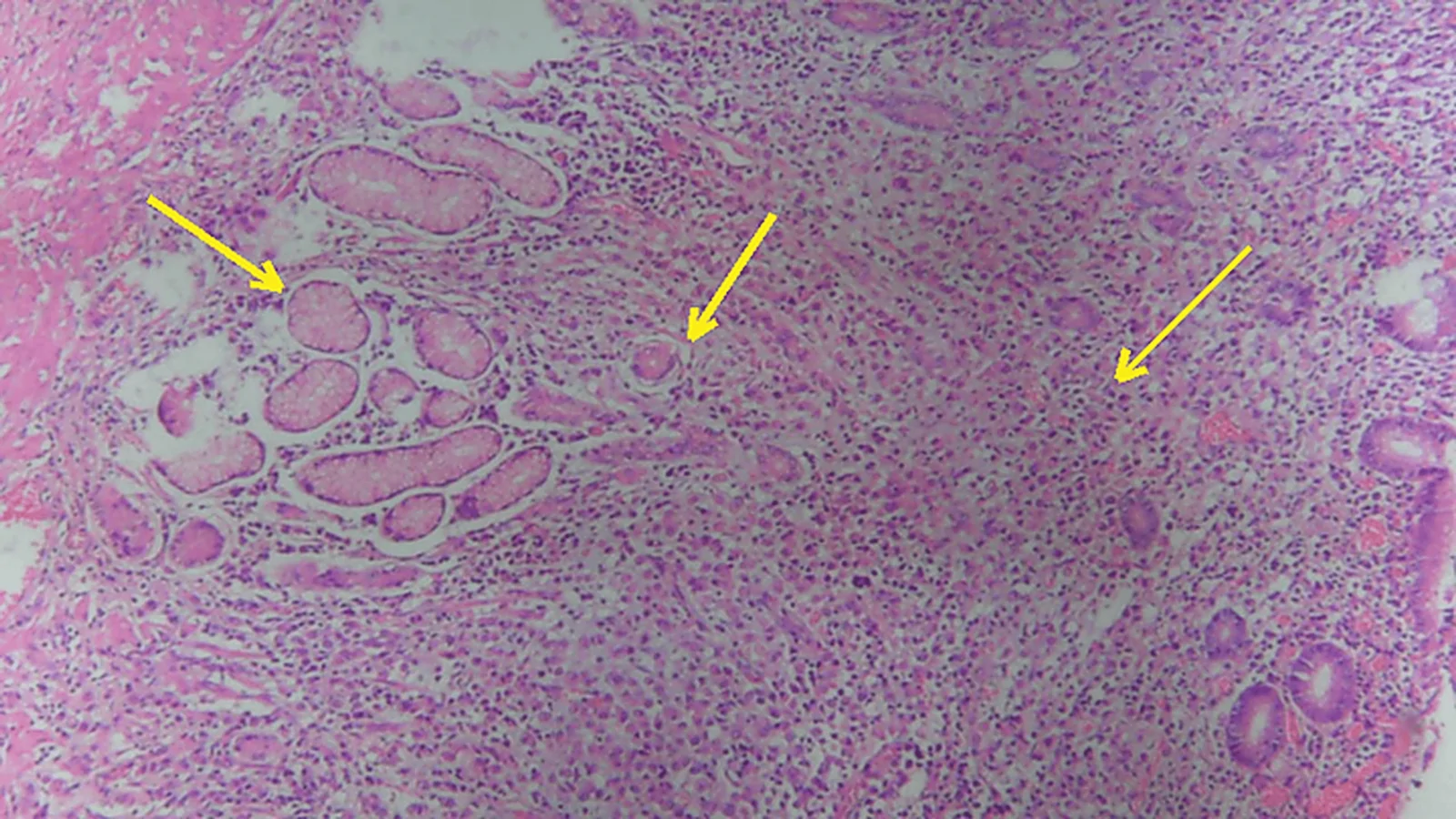
Low-power hematoxylin and eosin image (original magnification ×100) showing, from left to right along the yellow arrows, the transition from preserved gastric mucosa to infiltrating poorly differentiated adenocarcinoma

Gross and microscopic examination demonstrated a 1.2-cm poorly differentiated adenocarcinoma with signet-ring cell morphology, confined to the muscularis mucosae (Figures 8-11).

**Figure 12:**
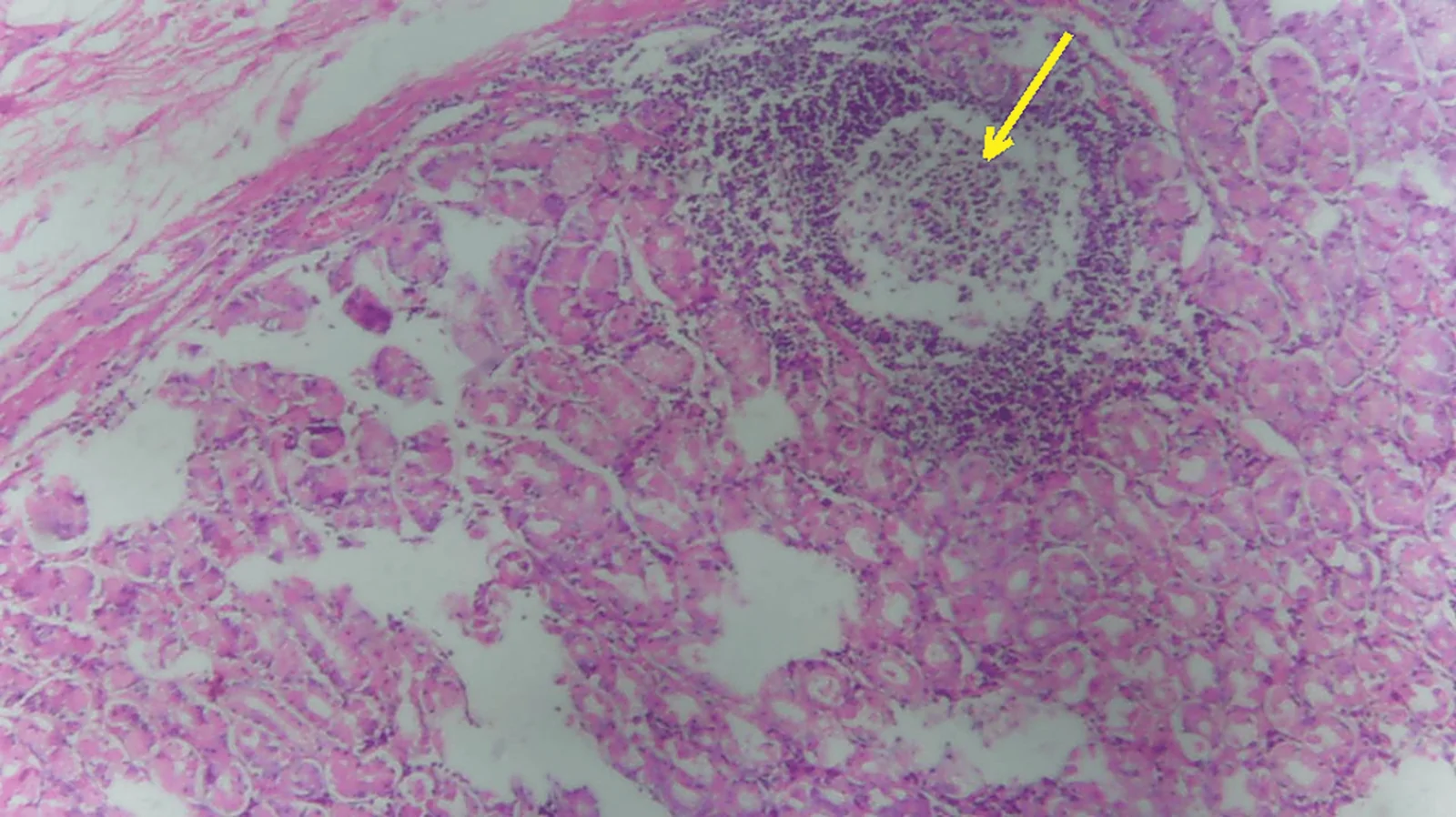
Hematoxylin and eosin image (original magnification ×200) showing a lymphoid follicle/aggregate (yellow arrow) and the presence of residual H. pylori *H. pylori*: *Helicobacter pylori*

At the most recent follow-up, approximately five months after surgery, the patient remained clinically well, without clinical or radiological evidence of recurrent adenocarcinoma or MALT lymphoma. Residual lymphoid aggregates were present adjacent to the carcinoma (Figure 12), and *H. pylori* remained positive. Twelve lesser omental and seven greater omental lymph nodes were negative for malignancy (0/19). The final pathologic stage was pT1aN0M0.

## Discussion

Synchronous gastric adenocarcinoma and MALT lymphoma have been recognized since the 1990s but remain uncommon. Wotherspoon and Isaacson described nine cases of adenocarcinoma associated with low-grade gastric MALT lymphoma, seven of which were *H. pylori*-positive [1]. Nakamura et al. subsequently reported synchronous or metachronous adenocarcinoma in patients with primary gastric lymphoma [4]. Lee et al. identified five synchronous cases in a clinicopathological series and showed that the two malignancies may arise at separate sites or form a collision lesion [2]. Reviews have documented only several dozen cases, whereas the specific combination of MALT lymphoma and signet-ring cell carcinoma has been reported only in isolated cases [3,8-10].

The association is probably underdiagnosed. MALT lymphoma may be multifocal and endoscopically subtle, appearing as erythema, erosion, depression, ulceration, enlarged folds, or infiltrative changes. A small carcinoma can be obscured by the dominant lymphoid process, and biopsy directed only at the most conspicuous area can miss the second neoplasm. Conversely, exuberant lymphoid infiltration around an adenocarcinoma may be dismissed as reactive. Careful gastric mapping, separate sampling of morphologically distinct areas, and integration of epithelial and lymphoid immunohistochemical markers are therefore essential [2,4]. Cytokeratin staining supports an epithelial component, while CD20, CD79a, BCL2, CD3/CD5, cyclin D1, Ki-67, and light-chain studies help characterize the lymphoid infiltrate and exclude mimics.

*H. pylori* is the most compelling shared etiologic factor. Chronic antigenic stimulation can induce the development of acquired gastric lymphoid tissue and clonal B-cell expansion. Additional genetic events, including t(11;18)(q21;q21)/BIRC3-MALT1, may confer antigen-independent growth and resistance to eradication therapy [5,6,11]. In parallel, chronic *H. pylori*-related inflammation increases the risk of gastric epithelial cancer. Population-level data also indicate an increased gastric adenocarcinoma risk among patients with gastric MALT lymphoma [12-14]. In the present patient, *H. pylori* was initially undetected but later remained demonstrable despite two treatment courses, supporting persistent infection as a common inflammatory background. The possibility of initial sampling error or reduced test sensitivity should be considered rather than assuming a newly acquired infection.

The preoperative EUS impression of superficial submucosal invasion contrasted with the final intramucosal stage. This discrepancy is clinically plausible. Ulceration and peritumoral fibrosis can appear hypoechoic and mimic tumor infiltration, thereby decreasing the accuracy of EUS and leading to overstaging, particularly in depressed or ulcerated lesions [13-15]. Accordingly, EUS depth should be interpreted together with histologic type, lesion size, ulceration, and high-quality endoscopic morphology rather than used in isolation. In this patient, an ulcerated undifferentiated-type cancer with suspected submucosal extension did not meet accepted endoscopic resection criteria, even though the resection specimen ultimately showed pT1a disease [13,16].

Management should be driven by the component with the greater oncologic risk while accounting for the extent and biology of both diseases. *H. pylori* eradication is first-line therapy for many localized gastric MALT lymphomas, with close endoscopic and histologic surveillance [5,17]. However, eradication alone would not adequately treat a synchronous, poorly differentiated signet-ring cell adenocarcinoma. Surgical resection was therefore appropriate in this case. Total gastrectomy was selected because of the previous antireflux operation, hiatal mesh, division of short gastric vessels, anticipated dense adhesions, and concern regarding the vascular reliability of a retained proximal stomach. These patient-specific considerations are more persuasive than the rarity of the tumor combination itself when explaining the extent of resection.

Robotic gastrectomy may facilitate precise dissection, suturing, and lymphadenectomy in technically demanding fields. Large comparative studies suggest lower blood loss and short-term oncologic outcomes at least comparable to those of laparoscopic gastrectomy, although longer operative time remains an important disadvantage [18-20]. This single case demonstrates feasibility but cannot establish superiority, improved survival, or generalizable safety. To our knowledge, this is the first reported robotic total gastrectomy performed specifically for synchronous gastric signet-ring cell adenocarcinoma and MALT lymphoma.

This report has limitations. It represents a single patient with short follow-up; the relative contribution of eradication therapy and gastrectomy to control of the MALT component cannot be separated; and molecular testing for clonality or MALT-associated translocations was not reported.

## Conclusions

Synchronous gastric signet-ring cell adenocarcinoma and MALT lymphoma are rare diagnostic and therapeutic challenges. Serial high-quality endoscopy, separate biopsies from all abnormal areas, and integrated histopathological and immunohistochemical assessment are central to preoperative recognition. *H. pylori* provides a plausible common inflammatory substrate, but treatment should be guided by the more aggressive tumor component. In this patient, robotic total gastrectomy enabled minimally invasive resection and D2 lymphadenectomy despite a previously operated, mesh-reinforced hiatus. The favorable postoperative course supports feasibility in a selected patient, while comparative effectiveness and oncologic superiority cannot be inferred from a single case.
